# Dosimetry Evaluation on the Use of ^18^F-FDG for PET/CT Imaging using OLINDA/EXM and Geant4 Monte Carlo Simulations – A Single Centre Experience

**DOI:** 10.21315/mjms-10-2024-800

**Published:** 2025-04-30

**Authors:** Muhammad Zulfadhli Zaini, Nurul Ab Aziz Hashikin, Aminudin Said, Hussin Dhalisa

**Affiliations:** 1 School of Physics, Universiti Sains Malaysia, Pulau Pinang, Malaysia; 2 Institut Kanser Negara, Putrajaya, Malaysia

**Keywords:** ^18^F-FDG, Institut Kanser Negara, IKN, Geant4 MC Simulation, OLINDA/EXM, ICRP, MIRD5 mathematical phantom

## Abstract

**Background:**

PET/CT using ^18^F-FDG has been increasingly used for diagnostic imaging. Thus, dosimetry evaluation is crucial to minimise unnecessary radiation exposure to other organs. This study aimed to evaluate the absorbed dose to patients undergoing ^18^F-FDG sequential PET/CT imaging in the Institut Kanser Negara (IKN), Putrajaya, Malaysia using OLINDA/EXM and Geant4 MC simulation, focusing on identifying the most suitable method for clinical application and analysing whether the absorbed dose complies with the safety standard set by ICRP 128.

**Methods:**

OLINDA/EXM (version 1.1) and Geant4 MC (version 10.6.0) software were used to evaluate the absorbed doses to the liver, kidneys, and urinary bladder. MIRD-5 mathematical phantom was used in Geant4 MC simulation, whereas in OLINDA/EXM, adult male and female models with organ masses normalised to the Japanese reference model were applied.

**Results:**

The mean absorbed doses to the liver, kidneys, and urinary bladder were 0.010 ± 0.006 mGy/MBq, 0.011 ± 0.004 mGy/MBq, and 0.025 ± 0.047 mGy/MBq, respectively, for OLINDA/EXM, and 0.009 ± 0.004 mGy/MBq, 0.011 ± 0.003 mGy/MBq, and 0.073 ± 0.086 mGy/MBq, respectively, for Geant4 MC. The corresponding differences between these two methods were 10.526%, 0%, and 97.959%, respectively for the liver, kidneys, and urinary bladder. The results comply with the ICRP-128 limits, indicating the safe use of ^18^F-FDG for diagnostic purposes in IKN.

**Conclusion:**

Both methods were shown to be reliable in estimating the absorbed dose for ^18^F-FDG. The selection of the methods for absorbed dose estimation depends on the capacity of the clinics (i.e., in terms of time and computational capability [power and literacy]).

## Introduction

In 2020, 16 million new cancer cases were reported, and according to the World Health Organization (WHO), 10 million deaths occur annually (1). The newly released Malaysia National Cancer Registry Report (MNCRR) 2012–2016 also found that the percentage of cancer cases detected in Stages 3 and 4 rose from 58.7% from 2007 to 2011 to 63.7% from 2012 to 2016. Fluorine-18 (^18^F)-fluorodeoxyglucose (FDG) used in positron emission tomography/computed tomography (PET/CT) has been increasingly used in nuclear medicine imaging. This modality has been proven effective for diagnosis, response evaluation, and recurrence detection in nuclear medicine (2). The use of ^18^F-FDG in PET/CT has been established in the diagnosis of patients since 2006 at the Institut Kanser Negara (IKN), Putrajaya, Malaysia. Although ^18^F-FDG is only used for diagnosis, it decays mainly through positron emission; thus, exposure to this particulate radiation may potentially lead to deterministic effects, such as cancer (3). Thus, dosimetry assessment is crucial to evaluate the exposure contributed by ^18^F on the target organs and other organs at risk (i.e., liver, kidneys, and urinary bladder) (4). However, there are currently limited published data available to address this.

Internal dosimetry is one of the important components of radiation safety for patients and radiation workers in radiological or nuclear facilities (4). Internal dosimetry involves assessing both the quality and the distribution of radiation energy deposited in bodily tissues by radioactive materials within the organism (5). Estimation of internal dosimetry requires information on the biokinetic data of the patients (6). Organ Level Internal Dose Assessment/Exponential Modelling (OLINDA/EXM) is one of the most widely used software for internal dosimetry (7, 8), which was developed by the Radiation Dose Assessment Resource (RADAR) as a replacement for the well-known Medical International Radiation Dose, MIRDOSE 3.1 (7). The software is useful for internal dose estimation, as it provides an EXM (7, 8) tool for the collection of time-activity data and also the integration of the data into a multi-exponential function to calculate the residence time.

Internal dosimetry can also be evaluated by simulating radiation doses using a method commonly referred to as Monte Carlo (MC) simulations (9). This simulation is considered the gold standard for dosimetry calculation, including internal dose estimation, although it requires some computational capability (10). The MC methods for dosimetry often employ simplified physical assumptions (9) to enhance computational speed (e.g., by assuming local energy deposition from low-energy electrons and photons); nevertheless, the methods may still require computational time ranging from 1 to 5 h to achieve acceptable statistical precision in all relevant organs. Several MC programmes (e.g., Geant4, MCNP, FLUKA, EGSnrc, and GATE) have been widely used for various dosimetry applications (11). The MC method can accurately simulate the geometry of the modality, as well as the interaction of radiation within the human body (12).

Thus, this study aimed to evaluate the absorbed dose to patients undergoing ^18^F-FDG sequential PET/CT imaging in the IKN using OLINDA/EXM and Geant4 MC simulation. Following data collection and analysis, the most suitable method for clinical application was determined between the two, and the absorbed doses obtained were assessed to determine whether the practice in the IKN complies with the safety standard set by ICRP-128.

## Methods

Ethical approval was obtained from the Medical Research and Ethics Committee, the Ministry of Health Malaysia prior to the start of this study (NMRR ID 22-01167-4DR [IIR]). This study was carried out between January 2021 and January 2022. The study involved using OLINDA/EXM and Geant4 MC simulation as a medium of analysing absorbed dose of ^18^F-FDG.

### Data Collection

This retrospective study included 30 adult patients (13 males and 17 females) with confirmed or suspected malignancies who underwent whole-body ^18^F-FDG PET/CT scans between January 2021 and January 2022. The scans were conducted after the MI-DR (GE Healthcare) PET/CT system had been fully installed and operational since October 2020. During this period, data from 1,619 patients who underwent ^18^F-FDG PET/CT scans were available for review. The minimum sample size required for the study was determined as 387, using a 95% confidence level and a 0.05 margin of error. However, due to specific inclusion and exclusion criteria, along with limitations associated with the newly installed PET/CT system at the IKN, the analysis was limited to 30 adult patients. To account for the reduced sample size, the confidence level was adjusted to 80% with a 0.05 margin of error, resulting in a required sample size of 27 patients. Consequently, the available sample size of 30 adult patients was sufficient for the study. Sample size calculations were performed using Minitab software (version 17.1.0). The mean age of the male patients was 58.85 ± 14.70 years, while the mean age of the female patients was 52.24 ± 12.34 years. The average weight of male patients was 70.04 ± 24.60 kg, and for female patients, the average weight was 67.98 ± 16.94 kg.

### Patient Criteria

The inclusion criteria of the PET/CT patients include adult patients who had received only ^18^F-FDG, suspected with metastatic disease, and underwent single and sequential PET/CT examination on the same day of appointment. Single PET/CT examination refers to a complete PET/CT examination, while sequential PET/CT examination refers to another (hence, sequential) PET/CT examination on the same day of appointment, which is also known as the “spot view”. The spot view scan involves a scan that only covers a certain scan region, such as the abdomen, abdomen to pelvis, thorax, thorax to pelvis, and pelvis. The exclusion criteria include patients who are pregnant, breastfeeding, diabetic, have a history of kidney failure, and are known to have allergy to intravenous contrast agents.

### Data Extraction

The extracted secondary patient data are listed in Table 1. The data were obtained from hardcopy documents and the PET/CT system. Medical Image Merge (MIM) software was used as an imaging tool after the patient images were retrieved from the infinite server. All the extractions were done by considering patients’ data confidentiality and were performed by the IKN nuclear medicine personnel.

### Dosimetry by OLINDA/EXM

**Figure 1:**
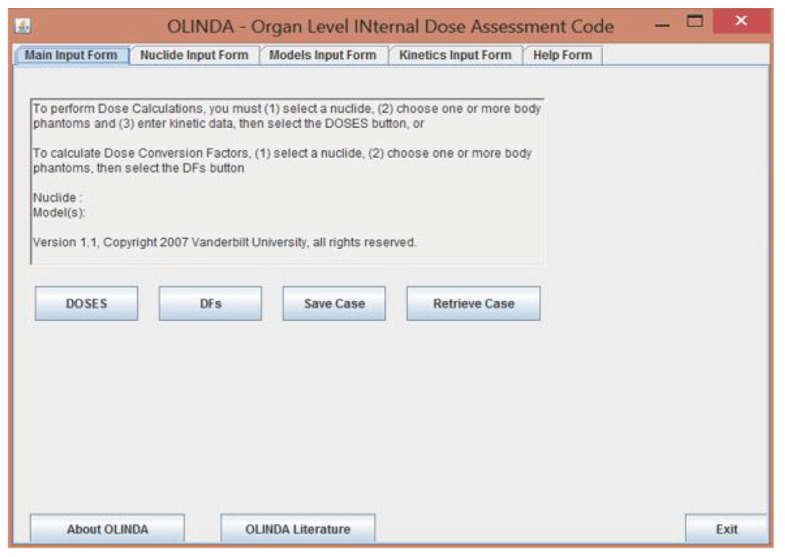
The main interface of OLINDA/EXM

OLINDA/EXM is an updated version of MIRDOSE 3.1 (7, 8), which is based on MC simulation and application of RADAR (13). All the updated information regarding nuclear medicine dosimetry can be searched online from the RADAR website. The main feature of OLINDA/EXM is shown in Figure 1.

**Figure 2:**
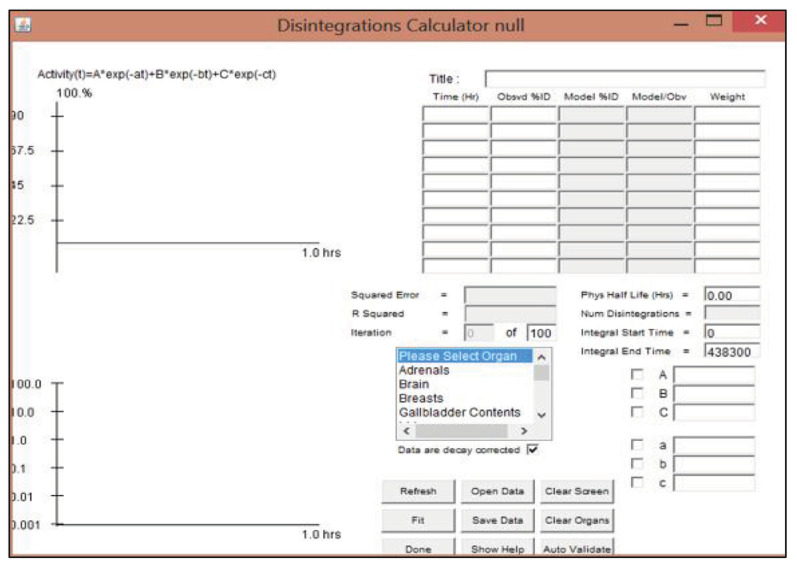
Fit data to model feature

The preferred appearance consists of the nuclide input form, model input form, and kinetic input form. OLINDA/EXM contains 300 radionuclides that are used in nuclear medicine (13), including 18F, which was chosen in this study. The number of disintegrations was manually entered by filling up the time point data at each organ, and “fit data to model” features were used, as shown in Figure 2.

**Figure 3:**
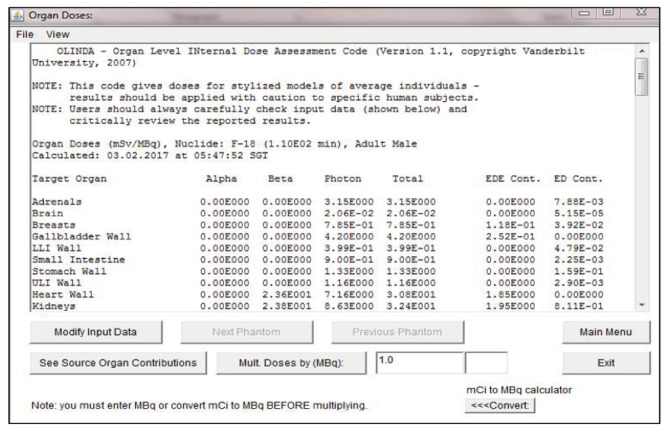
Example of interface results of total absorbed dose values on organs.

In this study, the absorbed dose was determined by the sum of radiation types (i.e., β and photon) in each internal organ. Figure 3 shows an example of the interface result of organ absorbed dose obtained from the OLINDA/EXM software. This study focused only on the liver, kidneys, and urinary bladder because only these organs show high uptake of radionuclides (14). Besides, the specific absorbed dose (SAD) values were derived by normalising the adult organ weights in OLINDA/EXM with references to the Japanese population (15).

### Dosimetry by Geant4 MC Simulation

MC simulations are an extensive application in radiological dosimetry that are employed to calculate the absorbed radiation dose within tissues and organs. The dosimetry procedure using MC simulations followed the American Association of Physicist in Medicine, AAPM 268, and Medical Internal Radiation Dose (MIRD) guidelines.

#### MIRD5 Phantom Geant4 MC Simulation

**Figure 4:**
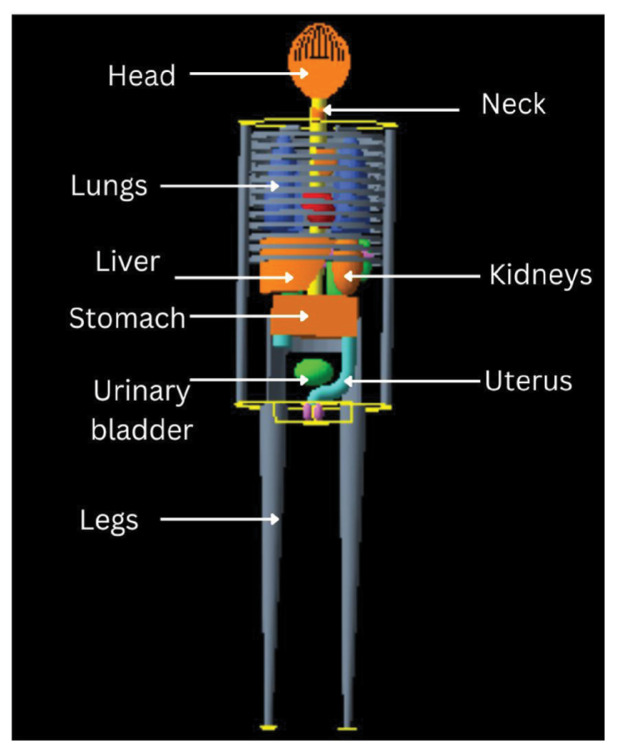
Mathematical hermaphrodite adult phantom

As outlined in the MIRD Pamphlet 5, this study utilised a mathematical hermaphrodite adult phantom, as shown in Figure 4, which is the first-generation mathematical phantom with all anatomical organs (16) that incorporates variations in body dimensions, shapes, and tissue compositions.

In this study, the Geant4 version 10.6 toolkit (17, 18) with an advanced example of human phantom was utilised, and only liver, kidneys, and urinary bladder were considered in this study. The original geometrical shape of organs from the mathematical MIRD-5 phantom was used as the simulation medium. The electromagnetic interactions of photons and electrons were modelled using the low-energy electromagnetic package (19), which is based on the Livermore Evaluation Atomic Data Libraries. The decay of ^18^F and its distribution in the liver, kidneys, urinary bladder, and other organs were simulated using the Geant4 radioactive decay and general particle source components (16), where the ^18^F was assumed to be uniformly distributed within these organs.

The organs’ activity was based on the percentage of uptake fraction relative to the injected activity determined using the MIM software. The simulation results were defined as the mean energy (MeV) deposited into each volume. The Geant4 MC package was employed to simulate the setup with 10^7^ histories. The simulation was performed three times for each parameter combination to achieve a standard deviation of less than 1% for the target organ (16). The absorbed doses (Gy) to the liver, kidneys, and urinary bladder were calculated by converting the MeV deposited to these organs to joules (J) and divided by the mass of each organ. The calculation for the number of histories is shown in Equation 1.


(1) $$\text{Number of histories},\text{N}={\text{A}}_{\text{o}}•{\text{f}}_{\text{h}}•1.44•{\text{T}}_{\text{e}}$$

Where A_0_ is the injected activity (MBq), T_e_ is effective half-life (h) (20), and f_h_ is the uptake fraction.

### Statistical Analysis

Statistical analysis was conducted using *t*-tests and Bland-Altman plots. These tests were used to determine the *P*-value and identify any outliers between the OLINDA/EXM and Geant4 MC simulation methods. The statistical analysis is important to evaluate the agreement, consistency, and reliability between the two methodologies, ensuring the validity of the results and identifying potential discrepancies in dose calculation approaches.

## Results

**Figure 5:**
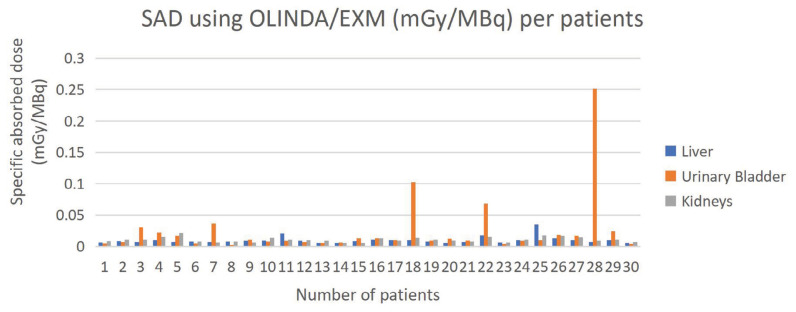
SAD using OLINDA/EXM

**Figure 6:**
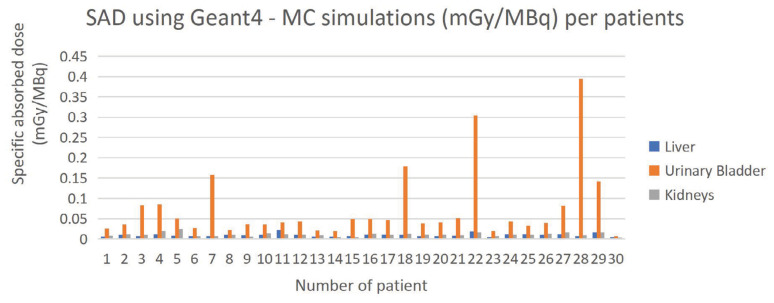
SAD using Geant4 – MC simulations

The urinary bladder received the highest SAD for both methods, followed by the liver. Kidneys recorded similar absorbed doses for both methods. The highest dose received by the urinary bladder was due to the accumulation of ^18^F-FDG (21). Figures 5 and 6 present the SAD values using OLINDA/EXM and Geant4 MC simulation. Each patient has a unique metabolism rate, which explains the reason for the differences in the SAD for each patient. The percentage difference in gender ranges from 11.43% to 89.04% for both methods (16).

Several factors contribute to the differences between these two methods, which will be discussed later in the subsequent section. The highest difference between the two methods was observed for the urinary bladder, followed by the liver. In contrast, kidneys demonstrated good agreement compared with the other two organs. Table 2 shows the percentage difference between OLINDA/EXM and Geant4 MC simulation.

**Figure 7:**
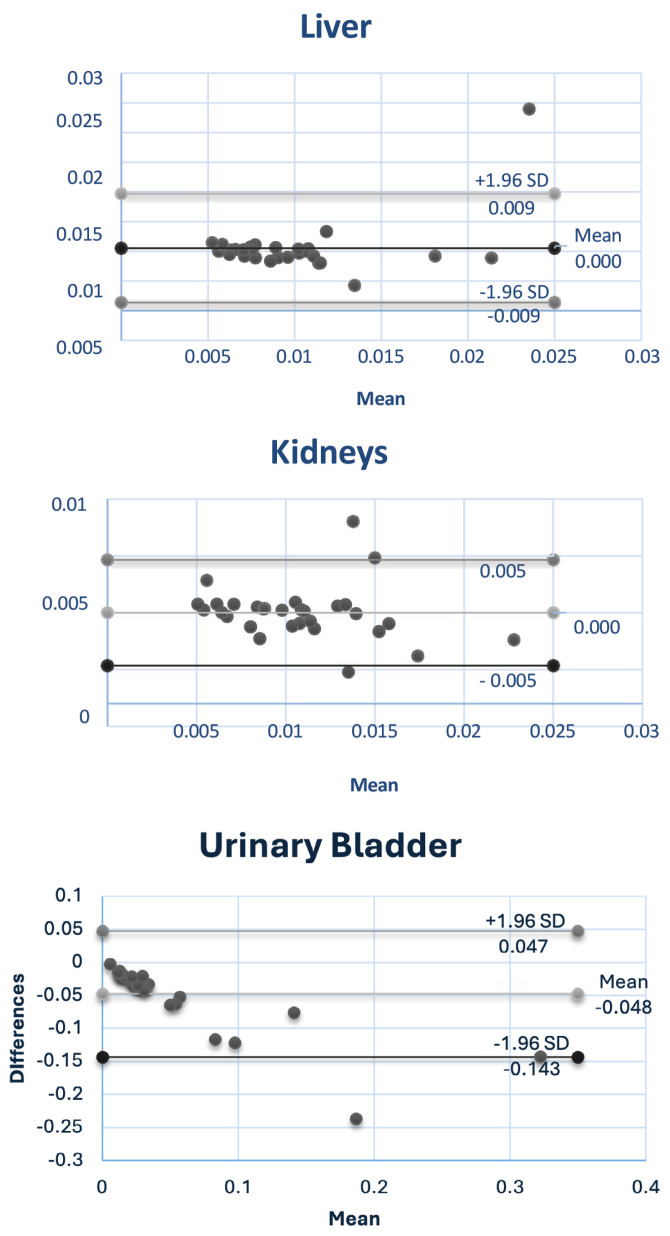
Bland-Altman plots for liver, kidneys, and urinary bladder.

Figure 7 depicts the Bland-Altman plots for the liver, kidneys, and urinary bladder. The data were analysed using a *t*-test to assess the significance of differences between the two methods. The *P*-values obtained for the liver, kidneys, and urinary bladder were 0.27, 0.49, and 0.00004, respectively. The urinary bladder showed a highly significant difference (*P* < 0.05), indicating high confidence that the observed difference is not due to random variation. Conversely, the liver and kidneys exhibited *P*-values greater than 0.05, suggesting no statistically significant differences between the two methods for these organs. This lack of significance may be attributed to individual patient variability, such as differences in biokinetics and organ volume.

## Discussion

Several factors may contribute to the slight differences between the results obtained from OLINDA/EXM and Geant4 MC simulation. The calculation method for determining the SAD significantly affects the absorbed dose values. For instance, OLINDA/EXM utilised the least square time point method, where only two time points were used due to the retrospective nature of this study, compared to other studies (22, 23) that employed multiple time points. Even in clinical practice, incorporating more time points generally yields a better fitting curve that represents the actual distribution of organ pharmacokinetics, and some studies (24) suggested that multi-exponential fitting may not consistently provide superior estimations of residence time. On the other hand, Geant4 MC simulation (17) assumed full exponential decay without relying on the use of time point measurements. Nonetheless, the statistical analysis shows good agreement between the two methods despite slight differences in the SADs.

Understanding the deformation characteristics of urinary bladder in dosimetry is essential for accurate radiation dose calculations. This deformation is caused by urine distribution, residual activity of ^18^F-FDG within the bladder, and physiological factors (e.g., bladder filling and emptying) (25). The deformation of the urinary bladder causes variations in the second time point of the least squared time point method in OLINDA/EXM compared to the estimation using Geant4 MC simulation, where this deformation is not considered. Although a dynamic bladder model that accounts for the deformation of urinary bladder has been shown to better estimate the SAD (24–25), in this study, neither method used the dynamic model. The deformation of urinary bladder (26) due to filling and emptying resulted in a high standard deviation in the estimated SAD.

Effective half-life of ^18^F-FDG is the duration for the radioactivity within the body to reduce by half due to the combination of physical and biological mechanisms. The factors affecting the effective half-life include physical half-life of the radiopharmaceutical, tissue uptake and metabolism, and renal clearance. The time-activity curve in OLINDA/EXM refers to the amount of injected activity (MBq) in certain organs from zero until time, *t*. In OLINDA/EXM, the effective half-life was calculated using the least square time point method; however, when using Geant4 MC simulation, the effective half-life was determined based on an earlier publication (20). This factor contributes to the differences in absorbed dose estimation when compared with OLINDA/EXM, where the biodistribution, metabolism, and excretion of the radiopharmaceuticals from the body were considered.

Both OLINDA/EXM and Geant4 MC demonstrated reliable absorbed dose estimation, in which the results were in good agreement between the two methods. The results showed comparable SADs for the liver and kidneys, where the differences were found to be less than 10%. In contrast, poor agreement was found for the urinary bladder. When contrasting OLINDA/EXM with Geant4.

MC simulations for absorbed dose estimation, the selection of the methodology depends on the particular applications and the desired degree of accuracy. While OLINDA/EXM offers a user-friendly interface, it is limited in terms of flexibility to simulate absorbed doses in various other clinical setups. Geant4, on the other hand, allows flexibility in simulating various dosimetry setups, but it is limited in terms of a user-friendly interface, especially in the clinical setting. Therefore, researchers and clinicians should carefully evaluate these considerations when determining the most suitable tool for their specific requirements. Nevertheless, based on the two methods, the absorbed doses recorded by these patients comply with the limits provided by ICRP-128 (i.e., 0.021 mGy/MBq, 0.017 mGy/MBq, and 0.130 mGy/MBq, for the liver, kidneys, and urinary bladder, respectively), indicating the safe use ^18^F-FDG for diagnostic purposes in our centre.

## Conclusion

Both OLINDA/EXM and Geant4 MC simulations have been applied in the estimation of absorbed doses in patients undergoing ^18^F-FDG in our centre. The results between the two methods were in good agreement for the liver and kidneys, while larger differences were noted for the urinary bladder. Although OLINDA/EXM offers a user-friendly interface, it is limited in terms of flexibility to simulate absorbed doses in various other clinical setups. Geant4, on the other hand, allows flexibility in simulating various dosimetry setups, but it is limited in terms of a user-friendly interface, especially in the clinical setting. Thus, the selection of the best dosimetry method should be balanced between both convenience and accuracy levels required by the centre. Nonetheless, the absorbed doses recorded in this study comply with the limits provided by ICRP-128, indicating the safe use of ^18^F-FDG for diagnostic purposes in our centre.

## Figures and Tables

**Table 1:** Extraction data from the PET/CT imaging and related documents

Patient data	Imaging tool data	Dosimetry tool data
Administered activity		Type of radionuclide
Physical characteristics (weight, age, etc.)	VOI based on Uptake activity (Bq/mL) and organ volume	Gender
		Age
		Kinetic data
		Organ mass
		Absorbed dose

Notes: VOI = volume of interest; Bq/mL = Baquerel/millilitres

**Table 2:** Percentage difference between OLINDA/EXM and Geant4 – MC simulations

Organ (*N* = 30)	OLINDA/EXM (mGy/MBq)	MC simulation (mGy/MBq)	Difference (%)
Kidneys	0.011 ± 0.004	0.011 ± 0.003	0
Liver	0.010 ± 0.006	0.009 ± 0.004	10.526
Urinary bladder	0.025 ± 0.047	0.073 ± 0.086	97.959
